# Elderly bedridden patients with dementia use over one quarter of resources in internal medicine wards in an Israeli hospital

**DOI:** 10.1186/s13584-020-00379-0

**Published:** 2020-05-01

**Authors:** Inbal Weiss Salz, Yehuda Carmeli, Avi Levin, Noga Fallach, Tali Braun, Sharon Amit

**Affiliations:** 1 National Center for Infection Control and Antibiotic Resistance, Tel Aviv Sourasky Medical Center, 6 Weizmann St. Tel Aviv, 64239 Tel Aviv, Israel; 2 Sackler Faculty of Medicine, Tel Aviv University, Tel Aviv, Israel; 3 Department of Epidemiology, Tel Aviv Sourasky Medical Center, Tel Aviv, Israel

**Keywords:** Geriatrics, Resource utilization, Antibiotic resistance, Mortality

## Abstract

**Background:**

Elderly bedridden patients with dementia (EBRPD) are a growing segment of the population. We aimed to describe acute care hospitalization of EBRPD in internal medicine wards: the prevalence of EBRPD, their impact on hospital resources and hospital ecology, one-year survival, and one-year readmission-free survival.

**Methods:**

The study setting was the internal medicine division of one tertiary care hospital in Israel. We conducted a point-prevalence survey to measure the prevalence of EBRPD and the prevalence of multidrug-resistant organism (MDRO) carriage. We also conducted a retrospective chart review of EBRPD who were hospitalized in the internal medicine division in order to assess resource use, survival, and readmission.

**Results:**

In the point prevalence surveys (*N* = 1667 patients), EBRPD comprised 24.3% of patients and 59.0% of mechanically ventilated patients. EBRPD were twice as likely to be colonized or infected by MDROs as other patients (39.3% vs. 18%, *p* < 0.001); thus, 41% of MDRO carriers during the survey days were EBRPD. In the retrospective study (*N* = 517 EBRPD), 80% of EBRPD received antibiotics; on average, they received an antibiotic on 87.7% of their hospital days. One-year survival was 35.6% and one-year readmission-free survival was 16.4%.

**Conclusions:**

Acute care hospitalization of EBRPD accounted for a high proportion of bed occupancy and ventilator use in internal medicine wards. EBRPD significantly increase the potential for MDRO transmission. Policymakers should seek alternatives to acute care hospitalization for EBRPD.

## Introduction

The prevalence of dementia increases with age. In OECD countries, dementia affects 2% of people aged 65–69, 7% of those aged 75–79, 20% of those aged 85–89, and 41% of people aged 90 and older [1]. The number of people living with dementia worldwide in 2011 was estimated to be 35.6 million and this number is expected to nearly double every 20 years, to 65.7 million in 2030 and 115.4 million in 2050 [2]. The estimated global cost of dementia in 2018 was 1 trillion US dollars [3]. Dementia ranges in severity from mild to severe cognitive impairment. Severe dementia is often accompanied by various levels of dependency, culminating in a bedridden state [4]. Bedridden patients with severe dementia are considered by many to have a poor quality of life that cannot be measured by the standard tools for calculating quality-adjusted life-years (QALYs), which are used to assess the value of medical interventions [5].

Patients with dementia are often admitted to acute care hospitals for treatment of infections or exacerbations of chronic conditions. The prognosis of patients with advanced dementia who are admitted to acute care hospitals is poor: studies have reported in-hospital mortality of 24% [6] and 6-month mortality of around 50% [7]. At the individual patient level, the overall contribution of acute care to quality of life and longevity has been questioned [8–13]. From the societal perspective, the hospitalization of elderly bedridden patients with dementia (EBRPD) in acute care hospitals carries a price, namely, a drain on scarce resources such as hospital beds. An additional societal toll is the ecological impact, i.e., the potential for collateral damage to other patients in the form of transmission of multidrug-resistant organisms (MDROs) and the use of antibiotics, which affect the hospital microbial ecology. These risks are not well described and have important ramifications for policy and clinical decision-making. Decisions about resource allocation in medicine are health care system-specific and reflect cultural values and preferences. However, often resources are allocated without systematic consideration of benefits, risks, and the overall burden on the health care system.

The Israeli healthcare system is characterized, on the one hand, by excellent access to care through universal national health insurance and a public hospital system offering advanced medical care, but, on the other hand, by a low number of hospital beds per 100,000 population [14], a high (95%) acute care bed occupancy rate [15], a shortage of ICU beds leading to use of mechanical ventilation in internal medicine wards [16], and low nurse-to-patient ratios [17]. As in most developed countries, the Israeli elderly population is rapidly growing. This raises serious policy questions about resource allocation in an overstretched hospital system.

The objective of this study was to explore the present state of the care of elderly bedridden patients with dementia in Israel. Specifically, we aimed: 1) to estimate the prevalence of EBRPD among patients hospitalized in the internal medicine division of a large tertiary care hospital; 2) to assess the impact of caring for EBRPD on hospital resources and on hospital ecology by measuring the intensity of care, the prevalence of MDRO carriage, and antibiotic usage; and 3) to report the one-year survival and one-year readmission-free survival of EBRPD hospitalized in the internal medicine division.

## Methods

### Setting

The study was performed in the internal medicine division of a 1500 bed tertiary care hospital in Israel. The internal medicine division has 400 beds, divided into 9 wards of 40–50 beds each. To compensate for a shortage of general ICU beds, each medical ward has an open-space step-up unit that is occupied by up to 8 severely ill patients, including those who require continuous monitoring, mechanical ventilation, and inotropic support.

### Study design. 1) point-prevalence survey

We conducted a point-prevalence survey to measure the prevalence of EBRPD and the prevalence of MDRO carriage in the internal medicine division. We performed the survey on 5 separate days between July and October 2013. The survey dates were spread out over 3 months, at least 2 weeks apart, to limit repeat counting of patients.

### 2) retrospective resource utilization study

We also conducted a retrospective electronic chart review of a convenience sample of EBRPD who were hospitalized in the internal medicine division between August 2012 and October 2013 in order to assess resource use, survival, and readmission. At that time, the hospital was transitioning, ward by ward, to electronic medical records. The sample comprised all EBRPD for whom full electronic medical records were available and who met the following definition of EBRPD: patients aged 65 and over with dementia, bedridden functional status, and complete immobility or severely impaired mobility (prior to hospital admission) according to documentation in the medical record. There were no exclusion criteria. In the point prevalence surveys, we consulted with the ward nurses to verify the EBRPD status of patients documented as such and found that the documentation was fully accurate.

### Data collection

For the point prevalence surveys, we collected the following data on all patients hospitalized on the survey days: age, sex, presence of dementia, whether the patient was bedridden, and mechanical ventilation. We reviewed reports from the microbiology laboratory to identify colonization or infection with the following MDROs: methicillin-resistant *S. aureus* (MRSA), vancomycin-resistant enterococci (VRE), carbapenem-resistant Enterobacteriaceae (CRE), extended-spectrum beta-lactamase- (ESBL) producing Enterobacteriaceae, *Clostridium difficile*, and multidrug-resistant (MDR) *A. baumannii*. For the retrospective study, we collected the following data from electronic medical records: age, sex, hospitalization or emergency department (ED) admission in the previous 6 months, length of stay, imaging studies performed (X-ray, ultrasound, CT), phlebotomy days, antibiotic days, and invasive procedures. Simple invasive procedures included insertion of a urinary catheter or nasogastric tube. Advanced invasive procedures included insertion of a percutaneous endoscopic gastrostomy (PEG) tube, lumbar puncture, surgery, mechanical ventilation, insertion of a central line, and insertion of a chest tube. One-year mortality was determined using the Israeli population registry (Ministry of Interior Affairs). One-year readmission and ED visits were determined using the hospital’s electronic admission and discharge database.

### Statistical analysis

For the point prevalence study, we used the chi square test and odds ratios to compare EBRPD to other patients. For the retrospective study, we constructed Kaplan-Meier curves of time to death and time to readmission or death. All analyses were conducted in Stata version 14.2 (Stata Corporation, College Station, Texas).

### Ethics statement

The study was approved by the hospital’s institutional review board. The requirement for informed consent was waived.

## Results

### Point prevalence study

On the 5 days surveyed, 1667 patients were hospitalized in the internal medicine division; 405 (24.3%) of them were classified as EBRPD. The proportion of patients who were EBPRD increased with age category: of the 443 patients over 85 years old, 204 (46.0%) were EBRPD. A high proportion of the mechanically ventilated patients were EBRPD: 95 out of 161 (59.0%). The prevalence of mechanical ventilation was 23.5% among EBRPD, compared to 5.2% among patients who were not EBRPD (*p* < 0.001). EBRPD were twice as likely to be colonized or infected by MDROs as other patients (39.3% vs. 18%, *p* < 0.001); thus, 41% of MDRO carriers during the survey days were EBRPD. Table 1 displays the odds ratios for colonization or infection by specific MDROs in EDBRP vs. other patients.

**Table 1:** Prevalence of colonization or infection with multidrug-resistant organisms in elderly bedridden patients with dementia vs. other patients

Organism	EBRPD (*n* = 405)	non-EBRPD (*n* = 1262)	Odds ratio (95% CI)	*P*
Vancomycin-resistant Enterococci	9 (2.2%)	20 (1.6%)	1.41 (0.64–3.12)	0.4
Methicillin-resistant *S. aureus*	50 (12.3%)	78 (6.2%)	2.14 (1.47–3.11)	< 0.001
Multidrug-resistant *A. baumannii*	35 (8.6%)	38 (3.0%)	3.05 (1.90–4.89)	< 0.001
Carbapenem-resistant Enterobacteriaceae	12 (3.0%)	32 (2.5%)	1.17 (0.60–2.30)	0.6
*C. difficile*	17 (4.2%)	21 (1.7%)	2.59 (1.35–4.96)	0.003
ESBL-producing Enterobacteriaceae	122 (30.1%)	137 (10.9%)	3.54 (2.69–4.67)	< 0.001
Any MDRO^a^	159 (39.3%)	227 (18.0%)	2.95 (2.30–3.77)	< 0.001

^a^A patient may carry multiple resistant bacteria

### Retrospective study

Characteristics of the 517 EBRPD in the study sample are presented in Table 2. Of these EBRPD, 273 (52.8%) had been admitted to the hospital or to the emergency department at least once in the previous 6 months, and they had a mean of 2.3 admissions (SD 2.5). Table 2 also summarizes the resources used during hospitalizations of EBRPD. All but 2 patients underwent imaging studies, with a mean of 3.7 studies per patient (SD 4.2). Blood tests were performed on 82.1% of patients’ days of stay on average (SD 23.7%). Eighty percent of patients received antibiotics and, on average, they received an antibiotic on 87.7% of their hospital days (SD 23.2%).

**Table 2:** Demographic and clinical characteristics and resource use during hospitalization of elderly bedridden patients with dementia

Characteristic	EBRPD (*N* = 517)
Age	
65–74	42 (8.1%)
75–84	155 (30.0%)
85+	320 (61.9%)
Male sex	222 (42.9%)
Hospitalization or emergency department visit in past 6 months	273 (52.8%)
Imaging studies, mean (SD)	3.7 (4.2)
Urinary catheter newly inserted	159 (30.8%)
Nasogastric tube newly inserted	108 (20.9%)
Advanced invasive procedures	100 (19.3%)
Hospital days with blood test performed, mean (SD)	82.1% (23.7%)
Antibiotic treatment	414 (80.1%)
Hospital days with antibiotic treatment, mean (SD)^a^	87.7% (23.2%)
Length of hospital stay (days), mean (SD)	9.4 (13.8)

^a^ Among patients who received antibiotics

**Fig. 1:**
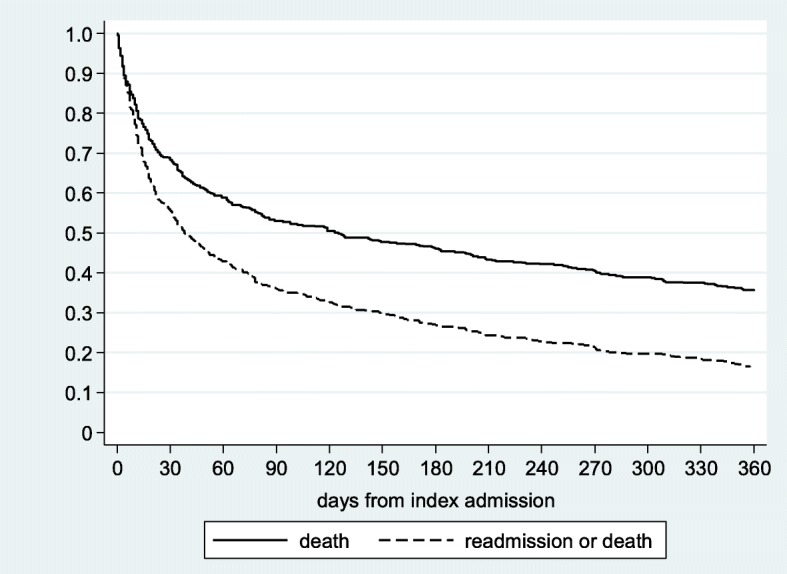
One-year survival and 1-year readmission-free survival among elderly bedridden patients with dementia

In-hospital mortality for EBRPD was 24.6%, and 184 patients (35.6%) survived 1 year from the date of admission. The median time until death was 125 days among the entire sample and 32 days among those who died within 1 year of admission. Of the 390 EBRPD who were discharged alive, 233 (59.7%) were readmitted to the hospital with 1 year of the index admission date. Figure 1 displays the 1-year survival and the 1 year readmission-free survival of EBPRD. Only slightly more than half of patients (55.5%) were alive and without readmissions at 30 days and 16.4% were alive and without readmissions at 1 year.

## Discussion

In this study, we aimed to provide data on acute care medical bed occupancy, ventilator use, and the ecological burden posed by hospitalization of EBRPD. The main findings of our study were: one quarter of beds in the internal medicine division were occupied by EBRPD. The intensity of care is high.

Similar to previous studies (7), we found that the prognosis of EBRPD hospitalized in acute care medicine wards was poor: half died within 4 months,and after 1 year only 16% of EBPRD remained alive and without readmissions. Because a high proportion of EBRPD are carriers of MDROs and are treated intensively with antibiotics, they may be a source for transmission of MDROs to others. Indeed, elderly patients admitted from long-term care settings have been often identified as the source of MDROs outbreaks in acute care hospitals [18, 19].

Hospitalization of a patient with dementia is a significant event that raises ethical dilemmas. From the individual patient perspective, acute care hospitalization is disruptive, inflicts discomfort and offers uncertain benefits in terms of longevity and quality of life. It has been argued that the treatment goals in EBRPD are to maintain quality of life, dignity and comfort [20], for which acute care hospitalization is not required. Israeli law permits advance directives for terminally ill patients but less than 0.3% of Israelis aged 65 and older have signed advance directive forms [21]. Cultural values and the low uptake of advance directives often lead to exhaustive treatment efforts, even when the prognosis and quality of life are poor [22]. Even advance directives do not guarantee a de-escalation of care, particularly antibiotic treatment. In a study of hospitalized Israeli patients in the last 2 weeks of life, 74% of those with advance directives (defined broadly as any documentation by the physician of patient preferences for end of life care) and 76% without advance directives received antibiotics [23]. From the societal perspective, there are two issues that need to be considered by policymakers: the widespread carriage of MDROs, which poses a risk to other patients, and the allocation of hospital beds, for which the competition is high. Among EBRPD there may be sub-populations with a better prognosis for whom acute care hospitalization may not be futile; characteristics of this group should be identified.

This study has several limitations. First it was a single center study; the prevalence of EBRPD in medical wards and their resources utilization likely varies between hospitals and between countries. Second, more detailed data describing the sample in the retrospective resource utilization study were not presented because of incomplete documentation.

## Conclusions

Our study highlights that acute hospitalization of EBRPD accounted for a quarter of all hospitalizations and more than half of mechanical ventilation use in internal medicine wards. Heavy antibiotic use and MDRO colonization among EBRPD pose an ecological threat. The prognosis of EBRPD was poor: only 36% survived for 1 year and only 16% survived and avoided re-hospitalization for 1 year. Policymakers must be aware of the magnitude of the use of medical ward resources by EBRPD and consider alternatives to acute care hospitalization, such as hospice and visiting nursing care at home (both underdeveloped in Israel). The public must be educated about advance directives.

## Data Availability

The data that support the findings of this study are available from Tel Aviv Sourasky Medical Center but restrictions apply to the availability of these data, which were used under license for the current study, and so are not publicly available. Data are, however, available from the authors upon reasonable request and with permission of Tel Aviv Sourasky Medical Center**.**

## Abbreviations

EBRPD: Elderly bedridden patients with dementia
MDRO: Multidrug-resistant organism
